# Epidemiology and risk factors for ovarian cancer incidence in the USA: a multilevel analysis

**DOI:** 10.7189/jogh.15.04354

**Published:** 2025-11-28

**Authors:** Victor Adekanmbi, Fangjian Guo, Jiefei Wang, Christine D Hsu, Thao N Hoang, Itunu Sokale, Yong-Fang Kuo, Olalekan Uthman, Abbey B Berenson

**Affiliations:** 1 Center for Interdisciplinary Research in Women's Health, School of Medicine, The University of Texas Medical Branch, Galveston, Texas, USA; 2 Department of Obstetrics and Gynecology, The University of Texas Medical Branch, Galveston, Texas, USA; 3 Department of Biostatistics and Data Science, The University of Texas Medical Branch, Galveston, Texas, USA; 4 Department of Medicine, Section of Epidemiology and Population Sciences, Baylor College of Medicine, Houston, Texas, USA; 5 Dan L. Duncan Comprehensive Cancer Center, Baylor College of Medicine, Houston, Texas, USA; 6 Division of Health Sciences, Warwick-Centre for Applied Health Research and Delivery, The University of Warwick, Coventry, UK

## Abstract

**Background:**

Ovarian cancer (OC) has the worst prognosis and highest death rate of all gynaecological cancers in the USA. We examined the independent effects of individual-, neighbourhood-, and state-level factors on ovarian cancer incidence using a multilevel analytical framework.

**Methods:**

In this retrospective cohort study, we analysed de-identified data from the All of Us research database, identifying women ≥18 years without prior ovarian cancer before January 2017. Participants were followed from 1 January 2017 through October 2023 (median follow-up: 6.6 years). Mixed-effects Cox regression models examined data on 85 388 individuals nested within ZIP-code areas and states, analysing individual-level risk factors and neighbourhood-level socioeconomic determinants, while accounting for geographic clustering. We fitted four progressive models: a null (random effects only), individual-level factors, neighbourhood-level factors, and full model with all covariates.

**Results:**

Among 85 388 women followed for a total of 569 847 person-years, 419 (0.49%) developed OC. Age demonstrated the strongest associations, with significantly elevated risks of developing OC among women aged 50–59 years (adjusted hazard ratio (aHR) = 1.83; 95% confidence interval (CI) = 1.28–2.61), 60–69 years (aHR = 2.01; 95% CI = 1.39–2.90), and ≥70 years (aHR = 1.67; 95% CI = 1.07–2.59) compared to those <40 years. Retired women had increased risk of OC compared to employed women (aHR = 1.39; 95% CI = 1.04–1.86). Non-Hispanic Black women demonstrated lower risk of OC than non-Hispanic White women (aHR = 0.63; 95% CI = 0.45–0.88). Regional variations showed 53% lower risk in the South *vs*. Northeast (aHR = 0.47; 95% CI = 0.25–0.86). Hormone replacement therapy was associated with increased risk of OC (aHR = 2.46; 95% CI = 1.07–5.67). Significant geographic clustering of OC was observed at neighbourhood and state levels.

**Conclusions:**

Individual-level factors, particularly age and employment status, are the primary determinants of OC risk, while apparent geographic disparities reflect population composition, rather than unmeasured environmental factors. The complete explanation of geographic clustering through measured covariates could provide important insights for targeted prevention strategies and future epidemiological research.

Ovarian cancer (OC) is the third most common gynaecological cancer after cervical and uterine cancer in the USA, where it is also the most fatal gynaecological malignancy and has the worst prognosis [1–3]. Available statistics indicate that about 23% of all gynaecological cancers are ovarian in origin, and more than half of those individuals newly diagnosed with OC yearly will die from this disease [2,4]. The American Cancer Society estimates that in 2025 alone, approximately 20 890 women in the USA will be diagnosed newly with OC, and 12 730 women will die from the disease [5].

Due to the lack of effective prevention strategies and accurate screening tests, most OC cases are detected at stages 3 and 4 (late stages) with associated poor prognosis and outcomes [6]. Despite recent treatment advances, studies have shown that the overall five-year survival rate from OC is below 50% [6,7]. In addition, the risk factors of OC are not well characterised, and its natural history is not well understood [8–10]. Therefore, large-scale population-based longitudinal data are needed to better understand the mechanisms underlying the natural history of the disease.

Several studies [11–17] carried out in the USA have examined factors predicting occurrence of OC in women aged 18 years and above. Many of them were conducted on individual-level compositional factors only (older age, family history of ovarian or related cancers, inherited gene mutations (like BRCA), obesity, smoking, endometriosis, and reproductive history), ignoring the contribution of contextual level factors (such as built environment, rural-urban residential pattern, healthcare access *etc*.). Presently, more attention is being paid to socioecological factors that directly or indirectly impact our health and health-related outcomes [18,19]. Concentrating on socioecological factors beyond individual compositional factors alone would be advantageous, as it would give us a clearer understanding of the contribution of social, economic, cultural and environmental factors to health and health-related outcomes.

To the best of our knowledge, no studies have examined the independent effects of individual and contextual/area-level factors on the incidence of OC in the USA. Examining both types of factors in the same analytical framework addresses limitations in methods seen when examining individual, neighbourhood, or state factors separately. Studies conducted using individual-level factors alone often will have elements of atomistic/individualistic fallacy stemming from inseparable relationships that individual and contextual level factors have with one another. Similarly, studies carried out with aggregate or area-level factors individually would lead to fallacy of division, as conclusions and inferences drawn from a cluster that an individual is a part of would not capture the distinctive characteristics of that individual that makes them to be different from other cluster members.

Our main aim was to determine the independent effects of individual-, neighbourhood-, and state-level contextual factors on predicting OC incidence in the USA.

## METHODS

### Participants and data management

We carried out this retrospective cohort study using a de-identified All of Us (AoU) research data record linked to American Community Survey data. The index date was defined as the start date (1 January 2017) of the study. All study subjects were required to have at least one clinic visit a year before and after the index date. We retrieved sociodemographic and behavioural variables (country of birth, employment status, marital status, physical and mental health indicators, smoking history, and annual income) from survey records and anthropometric measures from clinical measurements (height, weight, and standardised body mass index (BMI) fields), allowing classification of obesity (BMI>30). We identified relevant medical conditions, such as endometriosis, diabetes, and a history of specific genetic syndromes, from condition occurrence table. Participants were excluded if they had a documented history of OC prior to the study start date, and incident OC cases were determined by the earliest post-2017 date of diagnosis. We incorporated regional and socioeconomic information by linking ZIP code at the start of the study and state of residence records to external geospatial data sets.

Most variables in our data set had a relatively low proportion of missing data (~2%), except for annual income (~16%). We addressed these missing values using a random forest-based imputation method implemented in the ‘missForest’ package in *R*. This approach iteratively builds trees to predict missing values for both numeric and categorical variables until convergence is reached or a specified number of iterations is completed [20]. Character variables (*e.g.* country of birth) were first converted to factors, and the algorithm then estimated the most likely values based on existing information in the data set, resulting in a fully imputed data set for subsequent analyses. The OC variable was excluded from the imputation as it is the dependent variable.

In total, 65 479 out of 85 388 subjects had complete data. After the data imputation presented above, all 85 388 participants were retained as the study sample (Figure S1 in the **Online Supplementary Document**).

### OC case definition and diagnostic criteria

We identified incident OC cases after the index date (1 January 2017) using the AoU condition occurrence table based on International Classification of Diseases, 10th Revision (ICD-10) codes, as follows: C56 (malignant neoplasm of ovary), C57.0–C57.4 (malignant neoplasm of fallopian tube and other female genital organs), and D39.1 (neoplasm of uncertain behaviour of ovary). Only the first occurrence of any qualifying diagnosis was considered as the event date. We excluded cases with OC diagnoses recorded prior to 1 January 2017 to ensure incident cases only.

### Individual-level factors

We considered the following individual level factors: age, nativity, employment status, marital status, hormone replacement therapy (HRT), comorbid conditions, smoking status, and obesity prior to the index date (for diagnoses) or at the time of survey (for demographics). Our covariate selection was guided by established epidemiological literature on OC risk factors, including demographic factors (age, race/ethnicity), reproductive factors, medical history, and socioeconomic determinants. We categorised age groups as <40, 40–49, 50–59, 60–69, and ≥70 years based on established epidemiological patterns showing increasing OC incidence with age, particularly after menopause (typically around age 50), and clinical guidelines for cancer screening that use decade-based age categories. Annual income categories (USD ≤ 50 000, USD 50 000–75 000, USD 75 000–100 000, USD ≥ 100 000) were based on the USA Census Bureau median household income levels, while employment status was defined as employed, unemployed, and retired based on AoU survey responses, reflecting distinct socioeconomic and healthcare access patterns relevant to cancer risk. Smoking status was coded as non-smoker and smoker. Obesity was defined as BMI>30 kg/m2. Comorbid conditions of interest included pelvic inflammatory disease, endometriosis, diabetes, and Peutz-Jeghers syndrome, and were categorised as ‘yes’ or ‘no’. We likewise categorised HRT usage as ‘yes’ or ‘no’. Person-time was calculated for each participant from the index date until the occurrence of the event of interest, or the end of the study period, whichever came first. If no data are available on the index date, we used data from within a year prior to the index date.

### Neighbourhood-level factors

We obtained the percentage of high school graduates and households with assisted income within each patient’s primary ZIP code from the AoU socioeconomic status table, which is derived from the American Community Survey one-year estimates for 2017 provided by the USA Census Bureau. We linked the table to AoU data using ZIP code (aggregate data) as the unique identifier. Then, we categorised the percentage of high school graduates, houses that are vacant, households with assisted income, and households with no health insurance as quintile 1 (low), quintile 2 (low-middle), quintile 3 (middle), quintile 4 (high-middle) and quintile 5 (high). We used the term ‘neighbourhood’ to describe clustering within the same geographical living environment, basing it on sharing a common ZIP code area. Region of residence was categorised as Northeast, Mid-west, West, and South. We grouped ZIP code-level socioeconomic variables into quintiles (Q1–Q5) following standard practice in neighbourhood-effects research [21,22], providing equal sample sizes across categories while maintaining adequate power for detecting associations.

### Statistical analysis

We conducted a survival analysis using mixed-effects Cox proportional hazards models to examine associations between individual- and neighbourhood-level factors and occurrence of OC. Event time was calculated as the number of days from the index date (January 2017) to OC diagnosis. For patients without OC, survival times were censored at the date of their last clinic visit.

We specified four hierarchical models: the first (a null model with no explanatory variables) disintegrated the variations existing at the neighbourhood and state level, the second incorporated individual-level variables only, the third accounted for neighbourhood-level variables only, and the final model included all covariates with full adjustment. We estimated fixed effects for demographic variables (age, nativity, race/ethnicity, employment, marital status), health-related factors (obesity, smoking status, diabetes, pelvic inflammatory disease, endometriosis, HRT, and Peutz-Jeghers syndrome), and socioeconomic indicators (income, education, insurance coverage, housing stability and geographic region). Random intercepts at the state and zip code levels were included to account for geographic clustering. 

We used the variance inflation factor to check for multicollinearity. For each model, we reported adjusted hazard ratios (aHRs), variance components, intraclass correlation coefficient as well as median hazards ratios (MHRs) to assess influences of individual and contextual factors on the outcome of interest. We evaluated model fit using the Akaike information criterion (AIC), with lower values indicating improved explanatory power.

All analyses were performed using R, version 4.5.1 (R Core Team, Vienna, Austria).

#### Model convergence and stability, residual analysis, and model comparison

We assessed all mixed-effects Cox models for convergence using standard diagnostic criteria. Random effects variance components were estimated using restricted maximum likelihood methods. We verified model stability by examining parameter estimate consistency across different starting values and confirming positive definiteness of the variance-covariance matrix.

We examined martingale and deviance residuals to assess model fit and identify potential outliers or influential observations, and observed no systematic patterns in residual plots.

We used the AIC to compare nested models, with the full model (model 4) showing the best fit (AIC = 6772) compared to simpler alternatives.

### Ethical considerations

We analysed de-identified data sets collected between 2017 and 2023 by AoU research database. For this reason, our study was exempt from review by the University of Texas Medical Branch Institutional Review Board.

## RESULTS

**Figure 1:**
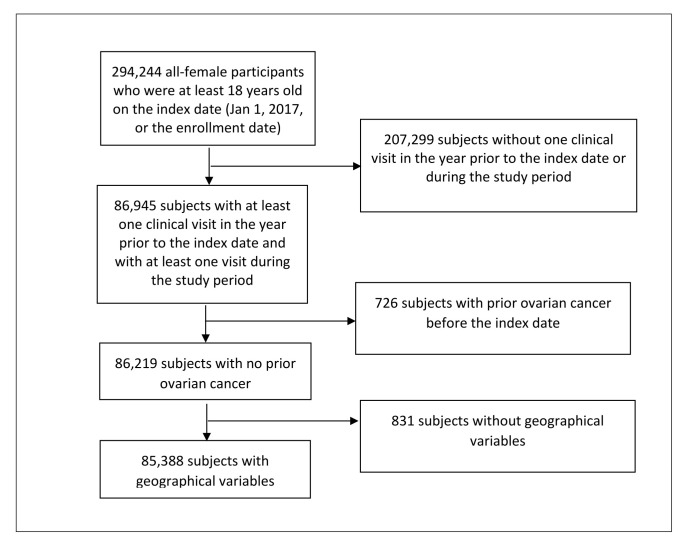
Flow diagram of participant inclusion and exclusion procedure.

From the retrieved sample of 294 244 female participants aged ≥18 years enrolled in the AoU research programme as of 1 January 2017 or their enrolment date, we excluded 207 299 (70.5%) participants who lacked the required clinical engagement (defined as having at least one clinical visit in the year prior to the index date and at least one visit during the study period), 726 (0.25%) with documented OC prior to the index date, and 831 (0.28%) without available geographical variables necessary for the multilevel analysis (**Figure 1**). The final analytic cohort of 85 388 (29.0%) women was followed from 1 January 2017 through October 2023, during which 419 participants (0.49%) developed incident OC.

**Figure 2:**
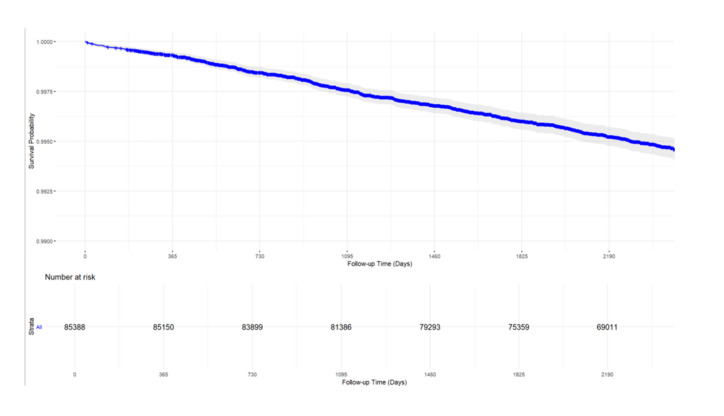
Kaplan-Meier survival plot with 95% CI.

As the Kaplan-Meier survival curve for the entire cohort over the study period shows (**Figure 2**), the survival probability declined from 1.0 at baseline to approximately 0.995 at 2190 days of follow-up. The number of participants at risk decreased from 85 388 at baseline to 85 150 at one year, 83 899 at two years, 81 386 at three years, 79 293 at four years, 75 359 at five years, and 69 011 at the end of follow-up (approximately six years). The median follow-up time for the entire cohort was 2421 days.

About 36% of those who developed OC were aged 60–69 years while 12% were aged <40 years (**Table 1**). There were significantly higher OC cases amongst retirees. Likewise, OC occurrence was highest among non-Hispanic white and individuals with more than USD 100 000 annual income. Furthermore, OC incidence was higher among individuals who resided in neighbourhoods with least proportion of high school graduates, on assisted income, no health insurance, and vacant houses. Similarly, those who resided in neighbourhoods located in Northeast region had highest incidence of OC.

**Table 1:** Characteristics of the study population*

	Ovarian cancer event			
	**Overall (n = 85 388)**	**No event (n = 84 969)**	**Event (n = 419)**	***P*-value†**
**Follow-up time, median (IQR), days**	2421 (2292–2452)	2423 (2297–2452)	1118 (593–1738)	<0.001
**Age group, years**				<0.001
*<40*	19 294 (22.6)	19 246 (22.7)	48 (11.5)	
*40–49*	13 397 (15.7)	13 351 (15.7)	46 (11.0)	
*50–59*	17 775 (20.8)	17 681 (20.8)	94 (22.4)	
*60–69*	22 032 (25.8)	21 883 (25.8)	149 (35.6)	
*≥70*	12 890 (15.1)	12 808 (15.1)	82 (19.6)	
**Country of birth**				>0.9
*US born*	79 461 (93.1)	79 070 (93.1)	391 (93.3)	
*Foreign born*	5927 (6.9)	5899 (6.9)	28 (6.7)	
**Employment status**				<0.001
*Employed*	41 298 (48.4)	41 139 (48.4)	159 (37.9)	
*Unemployed*	19 283 (22.6)	19 196 (22.6)	87 (20.8)	
*Retired*	24 807 (29.1)	24 634 (29.0)	173 (41.3)	
**Race/ethnicity**				<0.001
*White*	61 653 (72.2)	61 309 (72.2)	344 (82.1)	
*Black*	19 192 (22.5)	19 133 (22.5)	59 (14.1)	
*Hispanic*	1590 (1.9)	1585 (1.9)	5 (1.2)	
*Other/Unknown*	2953 (3.5)	2942 (3.5)	11 (2.6)	
**Marital status**				0.5
*Married*	46 684 (54.7)	46 447 (54.7)	237 (56.6)	
*Unmarried*	38 704 (45.3)	38 522 (45.3)	182 (43.4)	
**Obesity status**				0.4
*No obese*	48 921 (57.3)	48 672 (57.3)	249 (59.4)	
*Obese*	36 467 (42.7)	36 297 (42.7)	170 (40.6)	
**Smoking status**				0.2
*Non-smoker*	52 384 (61.3)	52 141 (61.4)	243 (58.0)	
*Ex-smoker/current smoker*	33 004 (38.7)	32 828 (38.6)	176 (42.0)	
**Diabetes**	13 437 (15.7)	13 366 (15.7)	71 (16.9)	0.5
**Pelvic inflammatory disease**	17 567 (20.6)	17 470 (20.6)	97 (23.2)	0.2
**Endometriosis**	2724 (3.2)	2703 (3.2)	21 (5.0)	0.047
**Hormone replacement therapy**	477 (0.6)	470 (0.6)	7 (1.7)	0.006
**Peutz-Jeghers syndrome**	167 (0.2)	164 (0.2)	3 (0.7)	0.062
**Annual household income in USD**				0.005
*≤50 000*	36 220 (42.4)	36 078 (42.5)	142 (33.9)	
*50 000–75 000*	12 967 (15.2)	12 896 (15.2)	71 (16.9)	
*75 000–100 000*	10 253 (12.0)	10 192 (12.0)	61 (14.6)	
*≥100 000*	25 948 (30.4)	25 803 (30.4)	145 (34.6)	
**ZIP code-level high school education quintile**				0.5
*Quintile 1*	17 126 (20.1)	17 031 (20.0)	95 (22.7)	
*Quintile 2*	17 045 (20.0)	16 968 (20.0)	77 (18.4)	
*Quintile 3*	17 095 (20.0)	17 004 (20.0)	91 (21.7)	
*Quintile 4*	17 496 (20.5)	17 419 (20.5)	77 (18.4)	
*Quintile 5*	16 626 (19.5)	16 547 (19.5)	79 (18.9)	
**ZIP code-level assisted income quintile**				<0.001
*Quintile 1*	18 236 (21.4)	18 134 (21.3)	102 (24.3)	
*Quintile 2*	16 523 (19.4)	16 451 (19.4)	72 (17.2)	
*Quintile 3*	17 038 (20.0)	16 958 (20.0)	80 (19.1)	
*Quintile 4*	16 532 (19.4)	16 446 (19.4)	86 (20.5)	
*Quintile 5*	17 059 (20.0)	16 980 (20.0)	79 (18.9)	
**ZIP code-level uninsured quintile**				<0.001
*Quintile 1*	18 438 (21.6)	18 317 (21.6)	121 (28.9)	
*Quintile 2*	15 958 (18.7)	15 899 (18.7)	59 (14.1)	
*Quintile 3*	18 167 (21.3)	18 071 (21.3)	96 (22.9)	
*Quintile 4*	15 779 (18.5)	15 721 (18.5)	58 (13.8)	
*Quintile 5*	17 046 (20.0)	16 961 (20.0)	85 (20.3)	
**ZIP code-level vacant housing quintile**				<0.001
*Quintile 1*	17 082 (20.0)	16 964 (20.0)	118 (28.2)	
*Quintile 2*	17 082 (20.0)	16 990 (20.0)	92 (22.0)	
*Quintile 3*	17 217 (20.2)	17 150 (20.2)	67 (16.0)	
*Quintile 4*	17 494 (20.5)	17 421 (20.5)	73 (17.4)	
*Quintile 5*	16 513 (19.3)	16 444 (19.4)	69 (16.5)	
**Geographic region**				0.009
*Northeast*	30 604 (35.8)	30 428 (35.8)	176 (42.0)	
*Midwest*	28 692 (33.6)	28 557 (33.6)	135 (32.2)	
*South*	16 342 (19.1)	16 285 (19.2)	57 (13.6)	
*West*	9750 (11.4)	9699 (11.4)	51 (12.2)	

*Values are presented as n (%) unless specified otherwise.

†Wilcoxon rank sum test or Pearson χ^2^ test.

### Multilevel analysis of the factors associated with risk of OC

The progression from the null model (model 1) to the full model (model 4) demonstrated substantial improvements in model fit (**Table 2**). Testing of the proportional hazard assumption identified violations for endometriosis (*P* = 0.039), ZIP-level uninsured proportion (*P* = 0.008), and ZIP code-level vacant housing proportion (*P* = 0.039). To address these violations, we used stratified Cox regression, allowing separate baseline hazards for each level of the violating variables while estimating effects for remaining covariates.

**Table 2:** Multilevel Cox regression model for rates of ovarian cancer in the USA*

	Model 1	Model 2	Model 3	Model 4
**Measures of associations (Fixed effects model**				
**Individual level factors**				
**Age in years**				
<40		ref		ref
40–49		1.23 (0.82–1.86)		1.24 (0.82–1.87)
50–59		1.83 (1.28–2.61)		1.83 (1.28–2.61)
60–69		2.01 (1.39–2.91)		2.01 (1.39–2.90)
≥70		1.67 (1.08–2.60)		1.67 (1.07–2.59)
**Nativity**				
US born		ref		ref
Foreign born		1.02 (0.66–1.56)		0.99 (0.64–1.52)
**Employment status**				
Employed		ref		ref
Unemployed		1.35 (1.01–1.80)		1.33 (1.00–1.78)
Retired		1.38 (1.03–1.85)		1.39 (1.04–1.86)
**Race/ethnicity**				
White, non-Hispanic		ref		ref
Black, non-Hispanic		0.65 (0.47–0.89)		0.63 (0.45–0.88)
Other/unknown		0.72 (0.30–1.77)		0.72 (0.29–1.76)
Hispanic		0.77 (0.39–1.50)		0.73 (0.37–1.43)
**Marital status**				
Married		ref		ref
Unmarried		1.12 (0.89–1.40)		1.09 (0.87–1.37)
**Obesity**				
No obese		ref		ref
Obese		1.03 (0.84–1.27)		1.03 (0.83–1.27)
**Smoking status**				
Non-smoker		ref		ref
Ex-smoker/current smoker		1.07 (0.88–1.31)		1.07 (0.87–1.30)
**Diabetes (yes *vs*. no)**		1.05 (0.80–1.37)		1.05 (0.80–1.37)
**Pelvic inflammatory disease**		1.09 (0.86–1.38)		1.10 (0.87–1.40)
**Hormone replacement therapy**		2.35 (1.07–5.16)		2.46 (1.07–5.67)
**Peutz Jeghers syndrome**		3.79 (1.21–11.81)		3.60 (1.15–11.25)
**Annual income in USD**				
<50 000		ref		ref
50 000–75 000		1.26 (0.93–1.72)		1.26 (0.92–1.72)
75 001–100 000		1.38 (0.99–1.94)		1.37 (0.97–1.92)
>100 000		1.35 (0.99–1.84)		1.31 (0.96–1.79)
**Neighbourhood-level factors**				
**Percentage of high school graduate**				
Quintile 5 (high)			ref	ref
Quintile 1 (low)			0.56 (0.36–0.89)	0.58 (0.37–0.91)
Quintile 2			0.80 (0.49–1.33)	0.76 (0.47–1.23)
Quintile 3			0.58 (0.32–1.04)	0.55 (0.31–0.97)
Quintile 4			0.53 (0.27–1.04)	0.48 (0.26–0.89)
**Percentage of assisted income**				
Quintile 1 (low)			ref	ref
Quintile 2			0.96 (0.62–1.48)	0.93 (0.61–1.40)
Quintile 3			1.18 (0.78–1.78)	1.23 (0.82–1.84)
Quintile 4			1.08 (0.68–1.70)	1.15 (0.74–1.77)
Quintile 5 (high)			0.87 (0.48–1.60)	0.94 (0.52–1.69)
**Region**				
Northeast			ref	ref
Mid-West			0.72 (0.44–1.19)	0.73 (0.50–1.06)
South			0.39 (0.19–0.79)	0.47 (0.25–0.86)
West			0.75 (0.38–1.48)	0.65 (0.39–1.08)
Measures of variations (random effects)				
State-level				
Variance (SD)	0.12 (0.35)	0.07 (0.27)	0.06 (0.24)	0.00 (0.00)
VPC (%)	3.6	2.2	1.7	0.0
MHR	1.40	1.29	1.26	1.01
Explained variance (%)	ref	40.3	53.2	99.9
Zip-code-level				
Variance (SD)	0.02 (0.15)	0.01 (0.09)	0.00 (0.02)	0.00 (0.00)
VPC (%)	4.3	2.4	1.7	0.0
MHR	1.16	1.09	1.01	1.00
Explained variance (%)	ref	67.7	99.0	100.0
Model fit statistics				
AIC	9415	9212	6967	6772
df	0	20	11	31

AIC – Akaike information criterion, df – degree of freedom, INC – intra-neighbourhood correlation, ISC – intra-state correlation, MHR – median hazard ratio, ref – reference, SD – standard deviation, VPC – variance partitioning coefficient

*Values are presented as aHR (95% CI) unless specified otherwise. Model 1 – null model. Model 2 – individual-level factors. Model 3 – neighbourhood factors. Model 4 – individual-level factors + neighbourhood factors.

Age emerged as the strongest predictor of OC incidence. Compared to women under 40 years of age, the risk of developing OC was significantly elevated among all older age groups in the final model (model 4). Women aged 50–59 years had an 83% increased risk of developing OC (aHR = 1.83; 95% confidence interval (CI) = 1.28–2.61), those aged 60–69 years had a two-fold increased risk of OC (aHR = 2.01; 95% CI = 1.39–2.90), and women aged ≥70 years had a 67% increased risk of OC (aHR = 1.67; 95% CI = 1.07–2.59). Women aged 40–49 years showed a non-significant 24% increased risk of OC (aHR = 1.24; 95% CI = 0.82–1.87). Employment status was significantly associated with OC risk after adjusting for all other factors. Compared to employed women, retired women (aHR = 1.39; 95% CI = 1.04–1.86) had significantly elevated risks of OC. Race and ethnicity showed significant associations, with non-Hispanic Black women having a 37% lower risk of developing OC compared to non-Hispanic White women (aHR = 0.63; 95% CI = 0.45–0.88).

For factors related to medical history, women with a history of HRT had a 2.5-fold increased risk of OC (aHR = 2.46; 95% CI = 1.07–5.67). Having Peutz-Jeghers syndrome was also associated with a 3.6-fold increased risk (aHR = 3.60; 95% CI = 1.15–11.25).

ZIP code-level educational attainment showed significant protective associations with OC risk. Compared to areas with the highest proportion of high school graduates (quintile 5), women residing in areas with lower educational attainment had significantly reduced risks: quintile 1 (lowest education, aHR = 0.58; 95% CI = 0.37–0.91), quintile 3 (aHR = 0.55; 95% CI = 0.31–0.97), and quintile 4 (aHR = 0.48; 95% CI = 0.26–0.89). Geographic region demonstrated significant variation in OC risk. Compared to the Northeast region, women residing in the South had a 53% lower risk of OC (aHR = 0.47; 95% CI = 0.25–0.86). The Midwest and West regions showed non-significant reductions in risk of OC compared to the Northeast.

### Measures of variation (random effects)

The multilevel analysis revealed significant geographic clustering in OC incidence at both state and ZIP-code levels, with progressive reduction in clustering as covariates were added to the models (**Table 2**). The null model (model 1) demonstrated modest but significant clustering at the state level, with a variance of 0.12 (standard deviation (SD) = 0.35) and a variance partition coefficient (VPC) of 3.6%. This indicates that 3.6% of the total variation in OC risk could be attributed to unmeasured state-level factors. The MHR of 1.40 suggests that if an individual moved from a state with low OC risk to a state with high risk, their hazard would increase by approximately 40%. The full model (model 4) incorporating both individual and neighbourhood factors explained 99.9% of the state-level variance, reducing it to effectively zero (variance <0.01, VPC = 0.0%, MHR = 1.01). This suggests that the measured covariates captured virtually all systematic state-level variation in OC risk.

ZIP-code level clustering was more pronounced in the null model, with a variance of 0.02 (SD = 0.15) and VPC of 4.3%, indicating that local neighbourhood factors accounted for a slightly larger proportion of variation than state-level factors. The full model (model 4) completely eliminated ZIP-code level clustering, explaining 100% of the local variance (variance <0.01, VPC = 0.0%, MHR = 1.00). This indicates that the combination of individual and neighbourhood covariates fully accounted for local geographic variation in OC risk.

We conducted a sensitivity analysis on dataset including individuals with complete data only. This did not significantly change the findings from our main analysis (Table S1 in the **Online Supplementary Document**).

## DISCUSSION

We examined the largest, most heterogeneous nationally representative longitudinal study population to date available in the USA to describe the epidemiology and factors associated with occurrence of OC. To our knowledge, our study is the first to examine the independent effects of individual and contextual factors on the incidence of OC in the USA in the same analytic framework. Both individual and contextual factors were found to be associated with OC incidence, with individual level factors having a greater influence.

Our results strongly confirm the well-documented age-related increase in OC risk [7,23,24], with women aged 50–59, 60–69, and ≥70 years having 83%, 101%, and 67% increased risks, respectively, compared to women under 40. The pronounced risk elevation beginning at age 50 aligns with the post-menopausal increase in OC incidence documented across multiple populations and supports current understanding of hormonal influences on ovarian carcinogenesis. The non-monotonic age relationship observed, with peak risks in the 60–69-year group rather than continuously increasing risk, may reflect several mechanisms including survivor bias (women susceptible to OC dying from other causes before age 70), an inverted U-shaped biological relationship where risk peaks during early post-menopausal years then declines, or competing mortality from other age-related conditions. Future studies should examine age-specific incidence rates in larger cohorts with longer follow-up and consider competing risk analyses to better understand the true age-OC relationship.

Several individual-level associations noted here represent new or underexplored findings. The 39% increased risk among retired women, independent of age, has not been widely reported and may reflect healthcare utilisation patterns, socioeconomic factors not captured by income, or unmeasured lifestyle characteristics associated with retirement status. The 37% lower risk among non-Hispanic Black women compared to non-Hispanic White women adds to emerging evidence of ethnic disparities in OC, though the mechanisms remain poorly understood.

The 53% lower OC risk in the South compared to the Northeast represents a substantial and previously underexplored regional variation that persisted after adjustment for individual and neighbourhood characteristics. This finding suggests either unmeasured environmental factors, regional differences in healthcare practices, or population characteristics not captured by our covariates. Counter-intuitively, women residing in neighbourhoods with higher educational attainment had increased OC risk, with 42–52% higher risks in the most vs least educated areas. This finding contradicts typical socioeconomic gradients in cancer and warrants further investigation. The complete elimination of geographic clustering after adjustment for measured covariates demonstrates that apparent spatial disparities in OC largely reflect systematic differences in population composition rather than unmeasured environmental exposures, providing important insights for cancer surveillance and aetiology research.

### Comparison with previous studies

We found a positive association between age of participants and incidence of OC in the USA, with older individuals being more likely to develop the conditions. A reasonable explanation for this finding could be that, as individuals age, the cells of their bodies become more fragile leading to more DNA damage over time, which increases mutations to build up that can result in cancer development [25,26]. Another plausible explanation could be because of many ovulatory cycles that older adults have had during their lifetime compared to less numbers in younger adults [27,28]. Regular ovulation has been shown to damage the lining of the ovaries, which increases the chances of mutations that can result in cancer [29,30]. Future population level screening for OC should probably start around age 50 years old.

Furthermore, we found a significant association between race/ethnicity and likelihood of occurrence of OC, whereby non-Hispanic black women were less likely to develop OC compared to non-Hispanic white. This may be related to higher rates of total abdominal hysterectomy with bilateral salpingo-oophorectomy that have been found among African American women compared to white women [31]. Available evidence suggests that prophylactic and therapeutic total abdominal hysterectomies alone, salpingo-oophorectomy alone or both combined reduce the risk of developing OC [32–35].

Our finding of association between OC incidence and HRT use supports the findings of similar studies [36,37] conducted in the USA and other developed countries. The biological mechanisms linking menopausal HRT use to OC occurrence is currently not well known. A plausible explanation could be that prolonged postmenopausal HRT use, especially oestrogen-only HRT promotes excessive proliferation and malignant transformation of ovarian cells [38,39]. Further studies are needed to fully understand the biological mechanisms between HRT and OC.

Our results indicating increased risk of OC incidence among high-income earners compared to low-income earners was unexpected. A reasonable explanation for this outcome, however, could be that most high-income earners have full health insurance coverage and will most likely exhibit high health seeking behaviour because they typically have greater access to quality healthcare and can afford preventive and risk lowering services including cancer screening (not currently recommended at population level). Having high health seeking behaviour and going for cancer screening services for those at high risk due to genetic factors may mean cancers are picked up earlier than in those who do not attend cancer screening services [40]. Screening tests, such as cancer antigen-125 blood tests and transvaginal ultrasounds, can however lead to false-positive results, which can cause unnecessary anxiety and lead to invasive procedures like surgery in those who do not actually have cancer. Another plausible reason could be because affluent people have access to HRTs (a known risk factor), and specialist facilities compared to low-income earners.

Our study highlights the effects of contextual-level factors on occurrence of OC in the USA, as the risk of developing OC was higher among residents of neighbourhoods in the Northeast region compared to those residing in neighbourhoods of Mid-west region. This result confirmed findings of previous studies [41,42] that showed the existence of regional differences in OC incidence in the USA. A plausible reason for this finding could be because of differences in availability of diagnostic equipment and facilities and accessibility to OC care services including screening services [41,42]. Most Northeast states have been noted to consist of many urban counties and zip code areas with greater proportion of large medical centres compared with states in other regions of the USA [43]. Thus, neighbourhoods in Northeast region are more likely to have well equipped cancer diagnostic centres which may mean that cancers are picked up more in this region than other regions.

### Implications for practice and future research

The robust age-related patterns observed here provide important guidance for clinical practice, supporting a risk-stratified approach to OC awareness and surveillance. The substantially elevated risk among women aged 50 years and older, with peak risk in the 60–69-year age group, reinforces current clinical guidelines that emphasise heightened vigilance for OC symptoms in post-menopausal women. Clinicians should maintain a particularly high suspicion for ovarian malignancy in women presenting with abdominal symptoms in this age range.

From a public health perspective, the complete elimination of geographic clustering after adjustment for measured individual and neighbourhood characteristics suggests that apparent regional disparities in OC incidence primarily reflect population composition rather than environmental exposures. This finding has important implications for cancer surveillance and resource allocation, indicating that public health interventions should focus on high-risk demographic groups rather than geographic targeting based on crude incidence rates.

The substantial regional variations observed, particularly the 53% lower risk in the South compared to the Northeast, warrant careful examination by public health officials and cancer registries. While our analysis suggests this may reflect surveillance bias rather than true etiologic differences, public health practice should investigate whether regional differences in healthcare infrastructure, specialist availability, or diagnostic practices contribute to these patterns.

The neighbourhood-level findings, particularly the counterintuitive association between higher educational attainment and increased OC risk, challenge conventional assumptions about socioeconomic health gradients and have implications for health equity initiatives. Public health programmes should avoid assumptions that affluent neighbourhoods necessarily have lower cancer burdens and should investigate whether increased healthcare utilisation in educated areas leads to enhanced case detection that masks true patterns in other communities.

The protective effect observed among non-Hispanic Black women represents a critical research priority that could inform prevention strategies for other populations. Future studies should employ comprehensive approaches including genetic analyses to identify protective variants, detailed reproductive history collection to examine cultural and behavioural factors, dietary pattern investigations to assess nutritional influences, and healthcare utilisation studies to understand access and screening patterns.

Future research should also focus on translating epidemiological findings into actionable prevention and early detection strategies. This includes developing and validating clinical risk prediction tools that incorporate the individual-level factors identified as significant predictors, investigating whether community-based interventions can address the neighbourhood-level disparities observed, and examining how healthcare system interventions can reduce regional variations in cancer detection and care quality.

### Strengths and limitations

This is one of the largest multilevel analyses of OC incidence conducted in the USA, with the sample of 85 388 participants providing substantial statistical power for detecting associations and examining geographic clustering patterns. The population-based design using the AoU research database offers important advantages in terms of demographic diversity and geographic representation across all 50 states. The multilevel analytical framework represents a significant methodological strength that allows simultaneous examination of individual, neighbourhood, and state-level factors while appropriately accounting for geographic clustering. This approach overcomes the limitations of single-level analyses that may suffer from ecological fallacy when using only area-level data or atomistic fallacy when examining only individual factors. The inclusion of neighbourhood-level socioeconomic data linked through ZIP codes provides a novel contribution to OC epidemiology, as few previous studies have examined area-level effects on OC incidence with such geographic breadth.

Our use of ZIP codes as neighbourhood proxies represents a significant limitation imposed by data availability in the AoU database, as they are known to be heterogeneous geographic units that may inadequately represent true neighbourhood environments. Smaller geographic units such as census tracts or block groups would provide more precise neighbourhood definitions, but these were not available for linkage with our cohort data. The retrospective cohort design using secondary data imposes limits. Temporal relationships for some exposures cannot be confirmed, and some important risk factors (*e.g.* reproductive history, family history, detailed medication use) were not comprehensively captured, raising the potential for residual confounding.

## CONCLUSIONS

This large-scale multilevel analysis of 85 388 women demonstrates that OC incidence in the USA is significantly influenced by individual demographic characteristics, with age representing the predominant risk factor. The two-fold increased risk among women aged 60–69 years and additional risk associated with retirement status provide clear targets for enhanced clinical surveillance.

## Additional material


Online Supplementary Document
