# Supplementary material for: Epidemiology and risk factors for ovarian cancer incidence in the USA: a multilevel analysis
Source: J Glob Health. 2025 Nov 28;15:04354. doi: 10.7189/jogh.15.04354 (PMC12659798; doi:10.7189/jogh.15.04354)
Supplement: Online Supplementary Document [file jogh-15-04354-s001.pdf]

Supplement to: Adekanmbi V, Guo F, Wang J, Hsu CD, Hoang TN, Sokale I, Kuo Y, Uthman O, Berenson AB. Epidemiology and risk factors for ovarian cancer incidence in the USA: a multilevel analysis. J Glob Health. 2025;15:04354.

Table S1: Multilevel Cox regression model for rates of ovarian cancer in the United States

Variable	Model 1[a]	Model 2[b]	Model 3[c]	Model 4[d]
**Fixed effects**	**HR (CI)**	**aHR (CI)**	**aHR (CI)**	**aHR (CI)**
**Individual level factors**				
**Age in years**				
<40		1.00 (reference)		1.00 (reference)
40-49		1.47(0.85-2.55)		1.48(0.85-2.56)
50-59		2.20(1.36-3.57)		2.22(1.37-3.61)
60-69		1.76(1.05-2.96)		1.76(1.05-2.95)
≥70		1.81(0.98-3.34)		1.78(0.97-3.30)
**Nativity**				
US born		1.00 (reference)		1.00 (reference)
Foreign born		1.03(0.58-1.86)		0.98(0.55-1.76)
**Employment status**				
Employed		1.00 (reference)		1.00 (reference)
Unemployed		1.46(0.99-2.17)		1.48(1.00-2.20)
Retired		1.90(1.27-2.85)		1.94(1.30-2.91)
**Race/Ethnicity**				
White, non-Hispanic		1.00 (reference)		1.00 (reference)
Hispanic		1.45(0.58-3.59)		1.32(0.53-3.29)
Other/unknown		0.92(0.37-2.27)		0.88(0.36-2.16)
Black, non-Hispanic		0.70(0.45-1.09)		0.66(0.42-1.04)
**Marital status**				
Married		1.00 (reference)		1.00 (reference)
Unmarried		1.33(0.98-1.80)		1.30(0.96-1.76)
**Obesity**				
No obese		1.00 (reference)		1.00 (reference)
Obese		1.09(0.81-1.45)		1.10(0.82-1.46)
**Smoking status**				
Non-smoker		1.00 (reference)		1.00 (reference)
Ex-smoker/Current smoker		1.18(0.89-1.55)		1.17(0.89-1.54)
**Diabetes**				
No		1.00 (reference)		1.00 (reference)
Yes		0.98(0.66-1.46)		0.98(0.66-1.45)
**Pelvic Inflammatory Disease**				
No		1.00 (reference)		1.00 (reference)
Yes		1.14(0.82-1.58)		1.12(0.81-1.56)
**Endometriosis**				
No		1.00 (reference)		1.00 (reference)
Yes		0.89(0.41-1.91)		0.89(0.41-1.91)
**Hormone Replacement Therapy**				
No		1.00 (reference)		1.00 (reference)
Yes		3.57(2.79-26.63)		4.39(3.81-33.11)

**Peutz Jeghers Syndrome**				
No		1.00 (reference)		1. (reference)
Yes		2.52(0.35-18.01)		2.59(0.36-18.54)
**Annual income**				
<$50,000		1.00 (reference)		1.00 (reference)
$50,000 - $75,000		1.65(1.08-2.52)		1.60(1.05-2.44)
$75,001 - $100,000		1.62(1.01-2.60)		1.56(0.97-2.51)
>$100,000		1.78(1.18-2.68)		1.68(1.11-2.53)
**Neighborhood-level factors**				
**Percentage of high school graduate**				
Quintile 1(Low)			1.00 (reference)	1.00 (reference)
Quintile 2			0.78(0.41-1.51)	0.76(0.40-1.44)
Quintile 3			0.70(0.33-1.48)	0.71(0.34-1.48)
Quintile 4			0.75(0.30-1.86)	0.77(0.32-1.87)
Quintile 5(High)			0.61(0.23-1.67)	0.63(0.24-1.62)
**Percentage of Assisted Income**				
Quintile 1(Low)			1.00 (reference)	1.00 (reference)
Quintile 2			0.84(0.48-1.45)	0.82(0.49-1.36)
Quintile 3			0.85(0.46-1.58)	0.95(0.52-1.74)
Quintile 4			0.92(0.49-1.72)	1.02(0.55-1.88)
Quintile 5(High)			0.76(0.33-1.78)	0.94(0.41-2.16)
**Percentage with no health insurance**				
Quintile 1(Low)			1.00 (reference)	1.00 (reference)
Quintile 2			1.38(0.76-1.50)	1.07(0.65-1.74)
Quintile 3			1.94(1.01-3.71)	1.38(0.83-2.32)
Quintile 4			1.87(0.79-4.44)	1.41(0.66-3.01)
Quintile 5(High)			1.83(0.66-5.05)	1.55(0.62-3.90)
**Percentage with vacant houses**				
Quintile 5(High)			1.00 (reference)	1.00 (reference)
Quintile 1(Low)			1.50(0.81-2.76)	1.53(0.85-2.75)
Quintile 2			0.97(0.54-1.76)	1.02(0.58-1.79)
Quintile 3			0.82(0.44-1.55)	0.82(0.44-1.52)
Quintile 4			0.74(0.39-1.41)	0.78(0.42-1.44)
**Region**				
Northeast			1.00 (reference)	1.00 (reference)
Mid-west			0.55(0.29-1.03)	0.60(0.40-0.92)
South			0.35(0.15-0.84)	0.42(0.20-0.92)
West			0.42(0.17-1.02)	0.43(0.22-0.84)
**Measures of variation**				
**State**				
Variance (SD)	2.72×10^{-1} (7.46×10^{-2})	2.59×10^{-1} (6.65×10^{-2})	3.77×10^{-3} (1.47×10^{-5})	3.86×10^{-3} (1.58×10^{-5})
Explained variation (%)	Reference	15.03	9.54	5.03
ISC, %	3.42	1.27	1.35	1.19
MHR	1.62	1.60	1.07	1.05
**Zip code**				

Variance (SD)	$3.41x10^{-1}$ ($1.09x10^{-1}$)	$2.72x10^{-1}$ ($7.19x10^{-2}$)	$1.36x10^{-2}$ ($1.96x10^{-4}$)	$4.92x10^{-3}$ ($2.49x10^{-5}$)
Explained variation (%)	Reference	28.65	16.01	19.10
INC, %	8.05	5.53	5.49	4.72
MHR	1.75	1.60	1.10	1.29
AIC	262164	235715	251335	227545

AIC; Akaike Information Criteria, SD; Standard Deviation, MHR; Median Hazard Ratio, INC; Intra-Neighborhood Correlation, ISC; Intra-State Correlation

Table S2. Adherence to JoGH's GRABDROP guidelines items

1. Please list all papers published by each co-author in previous 3 years that were based on secondary analysis of a big data repository

1. Polychronopoulou E, Raji MA, Kuo YF. Prescription Sequence Symmetry Analysis for Detection of Chronic Opioid Use Adverse Event Signals Using Administrative Claims Data. Pharmacotherapy. 2025 Nov 17. doi: 10.1002/phar.70081
2. Nguyen A, Kuo YF, Gao D, Raji M. Regional and Temporal Variation in Receipt of Gabapentinoid and SSRI/SNRI Therapy Among Older Cancer Survivors in the United States. Curr Oncol. 2025 Oct 17;32(10):576. doi: 10.3390/curroncol32100576
3. Wiysonge CS, Uthman MMB, Ndwandwe D, Uthman OA. Multilevel Analysis of Zero-Dose Children in Sub-Saharan Africa: A Three Delays Model Study. Vaccines (Basel). 2025 Sep 21;13(9):987. doi: 10.3390/vaccines13090987. PMID: 41012190; PMCID: PMC12474322
4. Patel M, Buchya MA, Uthman O. Ethnic-Specific Threshold Analysis and BMI and Waist Circumference Cutoffs for Cardiovascular Disease and Subjective Wellbeing: Results using Data from the UK Biobank. J Racial Ethn Health Disparities. 2024 Oct 11. doi: 10.1007/s40615-024-02193-9
5. Hoang TN, Berenson AB, Shan Y, Guo F, Adekanmbi V, Hsu C, Yu X, Kuo YF. Trends in HPV-associated cancer incidence in Texas medically underserved regions. Cancer Med. 2024 Aug;13(16):e70133. doi: 10.1002/cam4.70133
6. Adekanmbi V., Guo F., Hsu C., Gao D., Polychronopoulou E., Sokale I., Kuo Y., Berenson A.B. Trends in patients' characteristics, treatment, and outcomes of endometrial cancer in the United States, 2005-2020. Cancers. 2024; 16(7):1282.
7. Ijaiya MA, Anjorin S, Uthman OA. Quantifying the increased risk of illness in malnourished children: a global meta-analysis and propensity score matching approach. Glob Health Res Policy. 2024 Jul 31;9(1):29. doi: 10.1186/s41256-024-00371-0
8. Anjorin S, Okolie EA, Onuegbu C, Ijaiya M, Ayorinde A, Oyebode O, Uthman O. Neighbourhood effect and inequality in access to essential health services among mother-child paired samples: a decomposition analysis of data from 58 low- and middle-income countries. Int J Equity Health. 2024 Jun 28;23(1):130. doi: 10.1186/s12939-024-02194-4.
9. Adekanmbi V, Guo F, Hsu CD, Shan Y, Kuo YF, Berenson AB. Incomplete HPV Vaccination among Individuals Aged 27-45 Years in the United States: A Mixed-Effect Analysis of Individual and Contextual Factors. Vaccines (Basel). 2023 Apr 10;11(4):820. doi: 10.3390/vaccines11040820
10. Sokale IO, Thrift AP, Montealegre J, Adekanmbi V, Chido-Amajuoyi OG, Amuta A, Reitzel LR, Oluyomi AO. Geographic Variation in Late-Stage Cervical Cancer Diagnosis. JAMA Netw Open. 2023 Nov 1;6(11):e2343152. doi: 10.1001/jamanetworkopen.2023.43152

2. Please explain the key elements of your study design and the use of the available datasets that make your study an original scientific contribution

This study presents a comprehensive secondary analysis of All of Us data, examining the epidemiology and factors associated of incidence of ovarian cancer in the US using a mixed effects modelling approach. We conducted a survival analysis using mixed-effects Cox proportional hazards models to examine associations between individual- and neighbourhood-level factors and occurrence of OC. Event time was calculated as the number of days from the index date (January 2017) to OC diagnosis. For patients without OC, survival times were censored at the date of their last clinic visit. We specified four hierarchical models: the first (a null model with no explanatory variables) disintegrated the variations existing at the neighbourhood and state level, the second incorporated individual-level variables only, the third accounted for neighbourhood-level variables only, and the final model included all covariates with full adjustment. We estimated fixed effects for demographic variables (age, nativity, race/ethnicity, employment, marital status), health-related factors (obesity, smoking status, diabetes, pelvic inflammatory disease, endometriosis, HRT, and Peutz-Jeghers syndrome), and socioeconomic indicators (income, education, insurance coverage, housing stability and geographic region). Random intercepts at the state and zip code levels were included to account for geographic clustering.

3. Please list all publications that addressed similar research questions in the same dataset and indicate where you cited them in your paper

Mahoney DE, Mukherjee R, Thompson J. Elucidating the influences of social determinants of health on perceived overall health among African American/Black and Hispanic ovarian cancer survivors using the NIH All of Us Research Program. Gynecol Oncol. 2024 Oct;189:24-29. doi: 10.1016/j.ygyno.2024.06.027

4. Please explain how you addressed multiple testing through an appropriately rigorous statistical threshold and indicate this in the methods section

We tested the proportional hazards assumption using Schoenfeld residuals and the ‘cox.zph()’ function in *R*. The global test showed borderline significance ($P = 0.088$), with specific violations identified for endometriosis ($P = 0.039$), ZIP code-level uninsured proportion ($P = 0.008$), and ZIP code-level vacant housing proportion ($P = 0.039$). To address these violations, we used stratified Cox regression models, allowing separate baseline hazards for each level of the violating variables while estimating effects for remaining covariates. This approach maintains the validity of hazard ratio estimates for non-violating variables while appropriately handling time-varying effects.

5. Please declare to what extent have AI chatbots been used in developing your paper and to which parts of the paper did they contribute

AI chatbots were not used in the writing or analysis of this manuscript. All content was developed by the authors using manual literature review, statistical programming (R 4.5.1), and expert interpretation of results.
